# Supplementary material for: Label-free optical vibrational spectroscopy to detect the metabolic state of M. tuberculosis cells at the site of disease
Source: Sci Rep. 2017 Aug 29;7:9844. doi: 10.1038/s41598-017-10234-z (PMC5575044; doi:10.1038/s41598-017-10234-z)
Supplement: Supplementary file 1 — Supplementary Information [file 41598_2017_10234_MOESM1_ESM.pdf]

Label-free optical vibrational spectroscopy to detect the metabolic state of M. *tuberculosis* cells at the site of disease

Vincent O. Baron[1], Mingzhou Chen[2,*], Simon O. Clark[3], Ann Williams[3], Robert J. H. Hammond[1], Kishan Dholakia[2] and Stephen H. Gillespie[1,*]

[1] School of Medicine, University of St Andrews, St Andrews, UK, KY16 9TF

[2] SUPA, School of Physics and Astronomy, University of St Andrews, KY16 9SS, St Andrews, UK

[3]Public Health England, Porton Down, Salisbury, Wiltshire, SP4 0JG, UK

Supplementary data

Section 1. Single cell discrimination between lipid rich and lipid poor bacteria using WMR spectroscopy

We separated mixed cultures of *M. smegmatis, M. bovis* (Bacillus Calmette-Guérin, BCG) and *M. tuberculosis* into lipid rich (LR) and lipid poor (LP) fractions with greater than 90% purity using our previously published method [1], and performed WMR spectroscopy on the separated cells. We recorded Raman spectra from ~60 individual lipid rich (LR) and lipid poor (LP) mycobacteria cells (*M. smegmatis,* BCG and *M. tuberculosis*) *in-vitro*. The spectra from the mycobacterial species are illustrated in the **Supplementary Fig. 1** and also in **Fig. 2a** for *M. tuberculosis*. In wavelength modulated Raman spectra (WMR spectra) zero-crossings are equivalent to peak positions in standard Raman spectra and the peak-to-valley corresponds to the peak intensity in standard Raman spectra. If we examine the Raman spectra in a window from 1000 cm^{-1} to 1800 cm^{-1}, it contains the major point of differences between LR and LP for all species. The two phenotypes mainly differ in two lipid peaks at 1300 cm^{-1} (designated lipid band A) and at 1440-1450 cm^{-1} (designated lipid band B), see **Supplementary Fig. 1 a, c, e** (see **Supplementary Section 4** for the peak assignment). LR cells showed higher Raman peak intensity in both lipid bands (A and B) compared to LP cells. We subsequently used multivariate statistical analysis: Principal Component Analysis (PCA) [2] to treat all spectra from each of the subsets (LR cells and LP cells). All the principal components (PCs) obtained from PCA are orthogonal to each other while being arranged in a decreasing order accounting for the variation in the spectra. The first 7 PCs correspond to more than 70% of variance in all WMR spectra from

each phenotype subset. With only the first 3 PCs we can form a distinct cluster as seen in **Supplementary Fig. 1 b, d, f.** The two lipid phenotypes are distinguished with high specificity and sensitivity, *M. smegmatis* (93.8%/96.8%), *BCG* (100%/96.8%) and *M. tuberculosis* (92.6%/96.1%), when the first 7 PCs are used in the leave-one-out cross validation (LOOCV) for each cell subsets.

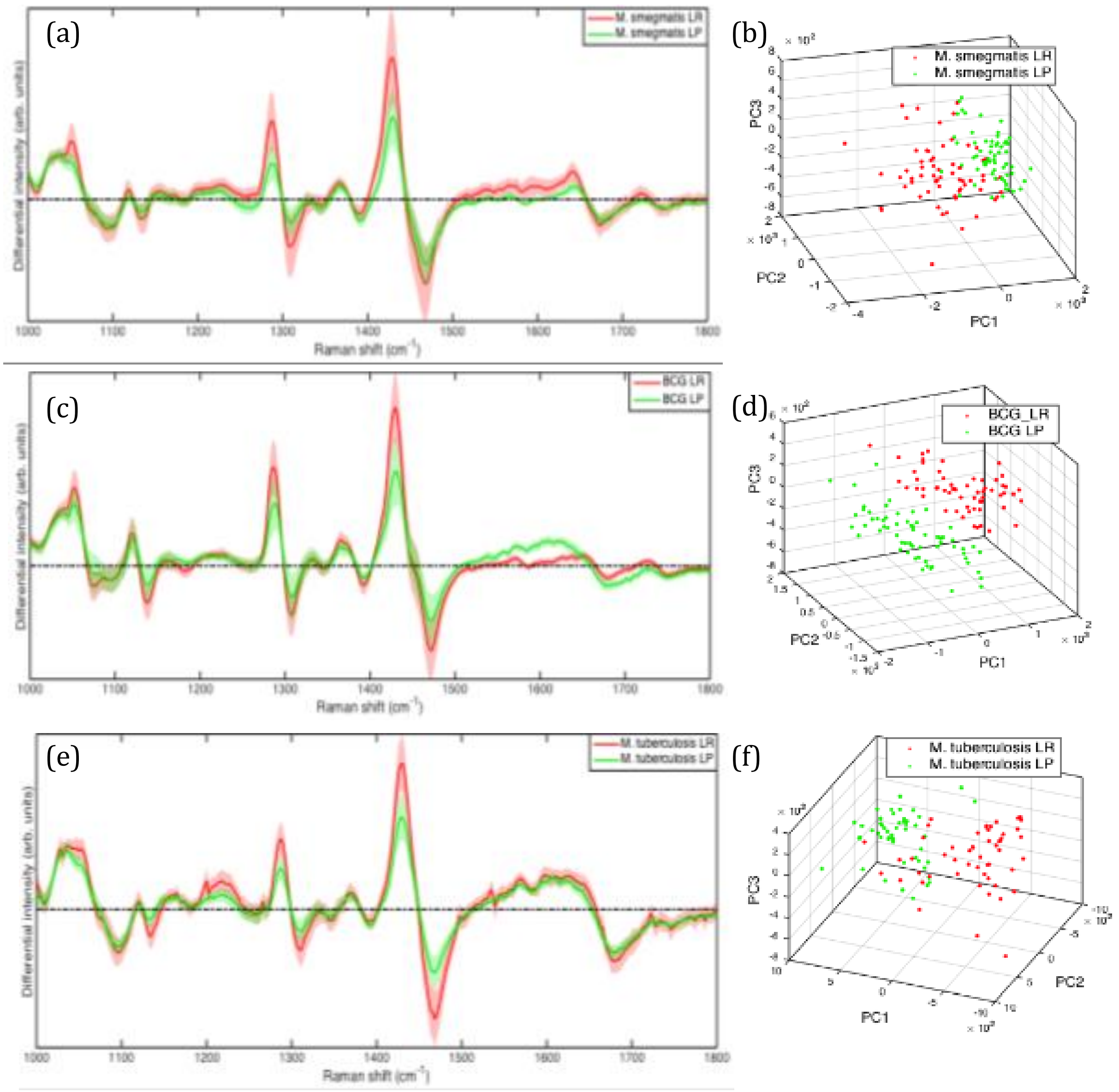

Supplementary Figure 1 *WMR spectra of M. tuberculosis, BCG and M. smegmatis phenotypes (LR and LP cells) and their corresponding PCA clusters. Mean spectra (**a, c, e**) of both LR (red curves) and LP (green curves) cells are calculated from ~60 WMR spectra taken from single bacteria. The colour-shaded area represents the associated single standard deviation. The x-coordinate in (**Fig. 1 a, c, e**) corresponds to the Raman shift (in wavenumber, cm^{-1}) and the y-coordinate corresponds to the differential Raman intensity in arbitrary units. In a WMR spectrum, a Raman peak is located at the zero crossing point (denoted by the dash-dotted line) while the Raman peak intensity is*

represented by the peak-to-valley value around that point. The acquisition time for each single bacterium is 150 seconds in total (see ***Methods section*** *for more details). Each dot in the 3D space (****b, d, f****) represents the first three PCs derived from the spectrum of single bacteria.*

Methods

Separation technique and control

An aliqot (2mL) of bacterial suspension were heat inactivated at 80°C for 20 minutes. The heat inactivation (80°C for 20 minutes) induces no significant modification in the Raman spectra [3]. The bacterial suspension was separated using a density based separation technique (1.04 $g.ml^{-1}$ D_2O/H_2O solution) similarly as previously described [1]. Both LR and LP fractions were stained using Nile red (Sigma-Aldrich) and viewed under a fluorescence microscope x100 (Leica DM5500) (excitation: 480/40 nm, 560/40 nm; emission: 527/30 nm, 645/75 nm) in order to understand the quality of the separation, the purity had to be greater than 90% over a 100 cell count to be validated.

LR and LP Sample preparation for Raman spectroscopy analysis

Separated cells fractions were re-suspended with 100 µl of PBS and 20 µl were placed on a thin quartz slide (SPI Supplies, 01015T-AB) left to air dry at 4 degrees and mounted on a thick quartz slide (SPI Supplies, 01016-AB) with a spacer filed with 15 µl of PBS. The bacteria were captured between the two slides in PBS. The mount was sealed with nail polish. This preparation was interrogated using Raman spectroscopy

Section 2. Raman data analysis using different Raman shift window show equivalent output

All data sets for *M. tuberculosis in-vitro* or in tissue are analysed with different Raman shift windows. The main differences between the two phenotypes are present in the two lipid bands A and B, which can be indicated by the increments in the variances from first 7 PCs in **Supplementary Tab1 to 3**. Therefore we still have good discrimination ability when only those two bands are chosen for analyses. The maximum standard deviations in sensitivity and specificity obtained using different Raman shift windows (in the region of 700 – 1800 cm^{-1}) is less than 0.044 in all our results for different samples as shown in **Supplementary Tab. 1 to 3**.

Supplementary Table. 1 *Specificity and sensitivity calculated using different Raman shift windows from WMR spectra for M. tuberculosis in-vitro. Standard deviations are 0.014 and 0.017 for sensitivity and specificity respectively.*

Raman shift window	Variance from first 7 PCs	Sensitivity	Specificity
700~1800 cm^{-1}	75.8%	0.96	0.91
1000~1800 cm^{-1}	78.1%	0.96	0.93
1000~1500 cm^{-1}	83.8%	0.98	0.93
1250~1500 cm^{-1}	89.8%	0.96	0.91
1400~1500 cm^{-1}	94.5%	0.94	0.89

Supplementary Table. 2 *Specificity and sensitivity calculated using different Raman shift windows from WMR spectra for stained M. tuberculosis in-vitro. Standard deviations are 0.018 and 0.028 for sensitivity and specificity respectively.*

Raman shift window	Variance from first 7 PCs	Sensitivity	Specificity
700~1800 cm^{-1}	80.5%	0.86	0.82
1000~1800 cm^{-1}	79.2%	0.84	0.80
1000~1500 cm^{-1}	83.5%	0.86	0.79
1250~1500 cm^{-1}	88.9%	0.86	0.85
1400~1500 cm^{-1}	93.7%	0.89	0.85

Supplementary Table. 3 *Specificity and sensitivity calculated using different Raman shift windows from WMR spectra for stained M. tuberculosis in tissue. Standard deviations are 0.022 and 0.044 for sensitivity and specificity respectively.*

Raman shift window	Variance from first 7 PCs	Sensitivity	Specificity
700~1800 cm^{-1}	78.9%	0.90	0.74
1000~1800 cm^{-1}	73.9%	0.93	0.85
1000~1500 cm^{-1}	79.4%	0.91	0.84
1250~1500 cm^{-1}	85.2%	0.87	0.79
1400~1500 cm^{-1}	92.9%	0.91	0.80

Section 3. Identification of *M.tuberculosis* LR and LP cells using a peak-to-peak ratio ($R_{\text{Band A/Ref band}}$)

A ratio $R_{\text{Band A/Ref band}}$ between the lipid Band A and the internal reference peak (1050-1070 cm^{-1}) was also investigated. Both LR and LP (from *in-vitro* culture) groups can be clearly identified (**Supplementary Fig. 2**). *M. tuberculosis* population acquired in the infected lung tissue show both LR and LP cells and the distribution is skewed towards high $R_{\text{Band A/Ref band}}$. This shows similar result than $R_{\text{Band B/Ref band}}$ as shown in **Fig. 2c** in the main text.

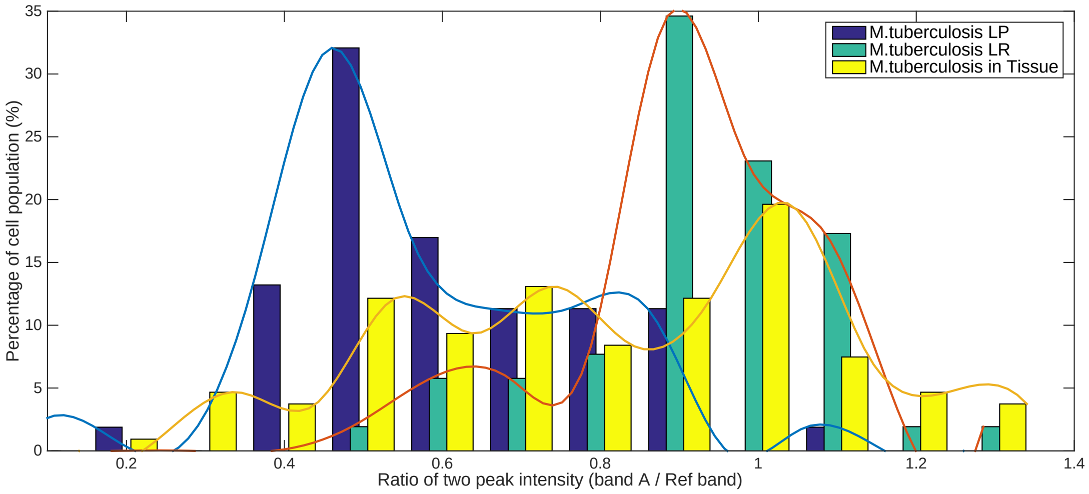

Supplementary Figure 2 *Percentage of M. tuberculosis in-vitro LR, LP and form tissue with a given peak-to-peak ratio (*$R_{\text{Band A/Ref Band}}$*) calculated by dividing the lipid band A intensity value by an internal reference band (Ref Band: 1050 cm^{-1} to 1070 cm^{-1}) peak intensity for each in-vitro M. tuberculosis WMR spectra. Due to the formalin and frozen procedure for the tissue, the peak intensities of lipid band A reduce to 61% of the peak intensities in the in-vitro population. This reduction in peak intensities in all ex-vivo Raman spectra will be taken into considerations when we calculate the lipid ratios between lipid band A and the non-lipid specific band (1050-1070 cm^{-1}). The x-coordinates correspond to the lipid ratio between 0.2 and 1.4 and the y-coordinates represent the percentage of the bacterial population for each specific lipid ratio value. The blue, green and yellow bars correspond to the in-vitro LP, the in-vitro LR and the ex-vivo populations respectively. Most of the LR population present a lipid ratio over 0.8 while the LP population has the lipid ratio less than 0.8. The curves show the envelops of these distributions.*

Section 4. Peak assignments

The major peaks observed in bacteria between 1000 cm^{-1} and 1800 cm^{-1} and their corresponding assignment are presented in the **Supplementary table 5**.

Supplementary table 5 *Peak assignments for the major peaks observed in bacteria in between 1000 cm^{-1} and 1800 cm^{-1}.*

Peak cm^{-1}	Assign bond	Chemical association	Reference
1000-1005	**C-C**	**Phenylalanine, proteins**	[4-7]
1050-1070 (Internal reference Band)	**C-C, C-N, C-O, PO_2^-**	**Proteins, lipids, DNA, RNA, carbohydrates, aromatic ring**	[4,5,7]
1125-1130	**C-C, C-N, C-O**	**Proteins, lipids, carbohydrates**	[4,5,8]
1230-1295	**Amide III**	**Protein**	[4,5]
1300 (lipid band A)	**CH2 twist vibration**	**Lipids**	[4,6-8]
1440 to 1450 (lipid band B)	**CH2, CH3 deformations**	**Lipids, fatty acids, proteins**	[4,6-8]
1650-1680	**C=C**<br>**Amide I**	**Lipids**<br>**Protein**	[7]<br>[8]
1735	**C=O**	**Lipids**	[5,7]

Section 5. Immunostaining

Frozen section of mice lymph node infected with BCG were attached on a positively charged glass slide or a positively charged quartz slide and then interrogated by WMR spectroscopy. Optimal Raman spectra can be achieved using quartz slides. A charged quartz slide was confirmed that it would not affect the Raman spectra (data not shown).

After WMR spectroscopy, the sample slide was used to perform immuno-staining, targeting T-cell with a CD3 primary antibody. Negative control did not show any specific staining on the cells, which means that the staining observed in the other slide was specific of T-cells. Similar results were observed when using any of the positively charged quartz or glass slide in terms of immuno-staining. Specific CY5 staining and good quality Hoechst counter-stain was observed. T-cells can be identified from other cells. Immuno-staining results from Nile red stained tissue and WMR spectroscopy interrogated tissue are shown in **Supplementary Figure 3** as a comparison. Immuno-staining has been successfully performed on tissue sample slides that have been interrogated by WMR spectroscopy without any difficulties. In contrast, the CY5 and Hoechst staining were poor in quality making very difficult to identify the T-cells once the tissue has been previously stained using Nile red. Therefore, Nile red staining procedure makes the immuno process much less efficient or even impossible.

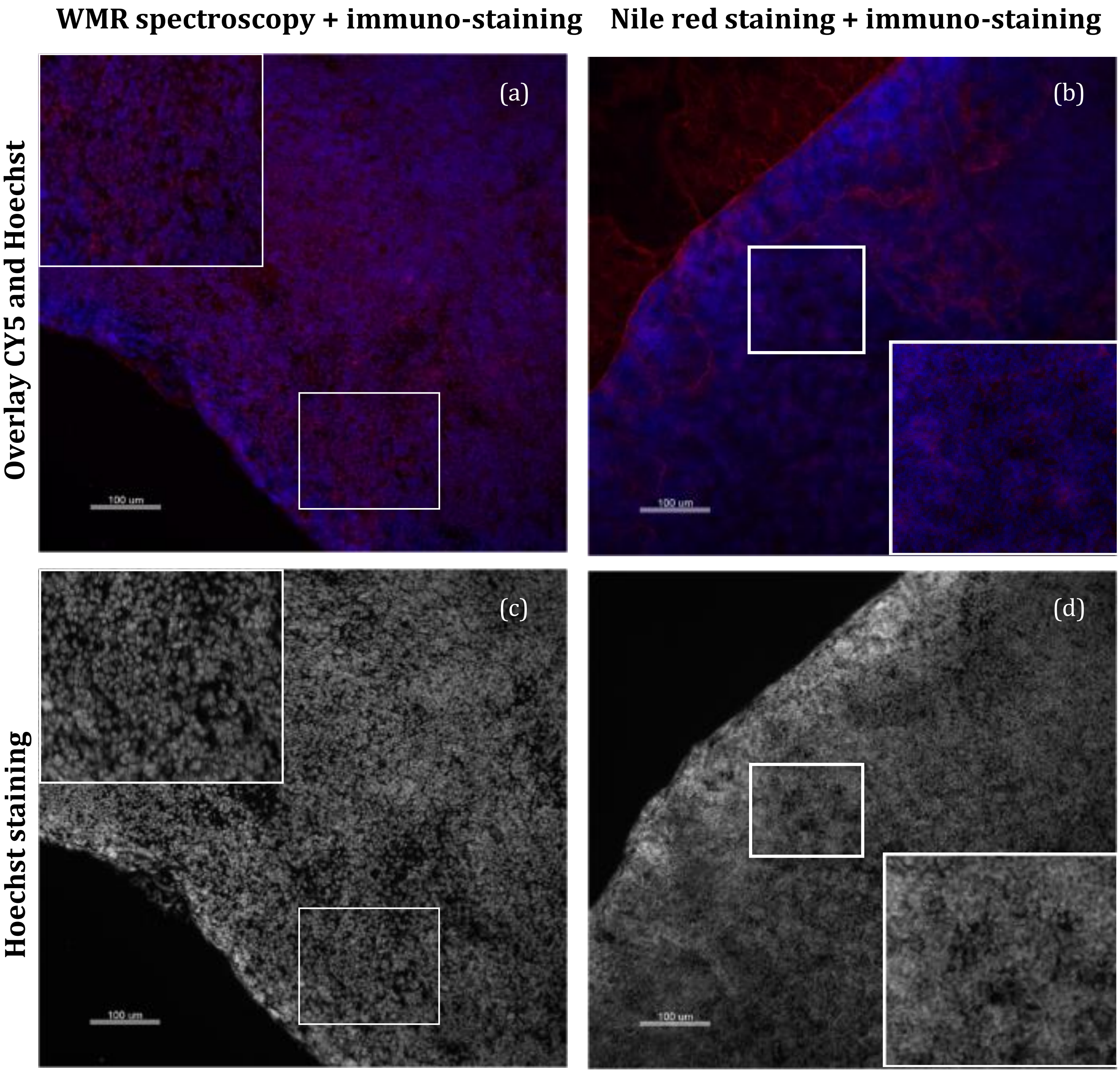

Supplementary Figure 3 *Comparison of immuno-staining procedure on tissue that have been previously interrogated by WMR spectroscopy (a, c) or Nile red staining (b, d). The first row shows the overlay pictures of CY5 (showing T-cells, red color) and Hoechst counter staining (showing the nuclei, blue color). The second row shows the Hoechst staining in gray scale. Insets at the corner of (a, b, c, d) show the enlarged image in the corresponding smaller rectangle.*

Based on the presented results we conclude that WMR spectroscopy and immuno-staining can be performed on the same sample and on the same slide though the slides need to be positively charged in advance. This charging process is fast, simple and costless by using Silane (Sigma) while it has no impact on the Raman spectra. Therefore, we confirm that using this label-free

optical method (WMR spectroscopy) does not modify the samples and allows further performing other techniques such as immune-histocytochemistry.

It is also clear that the immuno-staining procedure was strongly affected if the tissue sections were stained previously by Nile red. The CY5 emission was not specific showing lot of background and poor quality counter stain (Hoechst).

Methods

- **Protocol of positively charging quartz slides**

First the quartz slides were washed with distilled water and then with 70% ethanol. The slides were left to air-dry protected from dust for few minutes. In order to coat the slide, a 2% Silane solution was prepared using use 15 ml of acetone and 0.3 ml of 3-Aminopropyltriethoxysilane (Sigma A-3648). The quartz slides were dipped in Silane solution for 30 seconds. The slides were washed using distilled water for a few seconds and then left to air-dry at 37°C for several hours, protected from any potential dust.

- **Frozen section of Mice lymph node tissue**

Fresh BCG infected mice Lymph node tissue was stored at -80°C in the freezer. The preparing procedure was conducted on dry ice. First a sample mould was partially filled with OCT (Thermo Scientific) and the tissue was placed onto the mould before the OCT froze. Then another layer of OCT was added and left to freeze. A cryostat chuck was prepared by partially flooding the chuck with OCT and allowing it to freeze. The sample block was then removed from the mould and placed onto the chuck, over the frozen OCT layer. More OCT was added until the sample block was completely encased in frozen OCT. The chuck was placed on the cryostat (-20°C) and 10 micron thick sections were cut serially until the tissue was visible. The tissue sections were attached on positively charged slides (glass or quartz).

- **Attach tissue section to positively charged slides**

Three ways were investigated to attach BCG infected mice Lymph node frozen section on positively charged glass or quartz slides.

1. A quartz coverslip (SPI Supplies, 01015T-AB) attached with the tissue section was directly mounted on a thick quartz slide (SPI Supplies, 01016-AB) without any spacer or Phosphate-buffered saline (PBS).
2. A thick quartz slide (SPI Supplies, 01016-AB) attached with the tissue section was directly mounted on a quartz coverslip (SPI Supplies, 01015T-AB) without any spacer or PBS.

3. A glass microscopy slide (Fisher scientific, 10149870) attached with the tissue section was covered with a quartz coverslip (SPI Supplies, 01015T-AB) without any spacer or PBS.

The montage was then sealed by transparent nail polish at the corners.

- **Nile red Staining of frozen BCG infected lymph node section**

The 10-µm thick BCG infected mice lymph node frozen section were stained by Nile red (10µl of 25 $\mu g.ml^{-1}$ solution). The stained tissue was then washed with MiliQ water and left to air-dry. The tissue sections were covered by a coverslip and observed in Leica fluorescent microscope.

- **Procedure of immuno-staining**

After Raman interrogation or microscopic observation, the montage was opened and the slide attached with the tissue was stored at -20°C. Before the immuno-staining procedure, the incubations were carried out at room temperature in a dark humid box. The humidity in the box was kept by adding a wet piece of tissue in one side of the box.

The immuno-staining was performed as following: The slides were placed in PBST (PBS and tween 20 at 0.1%) for 10 minutes on constant agitation (35 oscillation per minute) in order to remove the OCT. A Dako pen (Dako, S2002) was used to draw on the slide an area around the tissue section to create a hydrophobic wall. A 3% H_2O_2 solution was prepared from a 30% stock solution (Sigma, H1009) with distilled water. 120 µl of this 3 % H_2O_2 solution was added on top of the tissue sections and left incubation for 10 minutes. The slides were then placed in PBST for five minutes under constant agitation (35 oscillation per minute). Two drops of Dako serum-free protein block (Dako, X0909) were added on the tissue sections and left incubation for 10 minutes. In the mean time the primary antibody (Dako, A0452, CD3 antibody) was prepared by diluting it, as 1 in 400, in Dako (S0809) solution. Without a wash, 120 µl of primary antibody were added on top of the tissue and left incubation for 30 minutes. The slides were then washed twice in PBST for 5 minutes under the same constant agitation. Three drops of the secondary antibody (Dako, K4003) solution was added on top of the tissue sections and left incubation for 30 minutes. The slides were then washed twice in PBST for 5 minutes under the same constant agitation. 120 µl of CY5 (PerkinElmer, NEL745 B001KT) was put on top of the tissue sections and left incubation for 10 minutes. The slides were washed twice again in PBST for 5 minutes under the same constant agitation. A 120 µl of Hoechst (Thermo Fisher, H3570) stain solution diluted at 1:1000 in distilled water was added on top of the tissue and left incubation for 10 minutes. The slides were washed twice in PBST for 5 minutes each time under the same constant agitation. After the second wash the slides were directly placed in 80% ethanol for one minute and left to air-dry for 10 minutes in darkness. Once dry the slides were mounted between a slide and a coverslip

within 30 µl of mounting medium (Life technology, P36930). The slides were left to dry for few hours at room temperature and stored at 4°C. The slides were observed in HistoRX microscope station (HistoRX, PM2000) using the AQUA software. The settings used were 2 ms acquisition time for Hoechst and 30 ms for CY5.

Section 6. Impact of Nile Red staining on the WMR spectrum of single bacterium

The impact of Nile red staining on the WMR spectrum of single bacterium was investigated. A culture of *M.smegmatis* was stained using Nile Red (2.5 µg.ml^{-1}). The stained bacteria and unstained *M.smegmatis* cells were interrogated by WMR spectroscopy and their spectra compared. The result is shown in the **Supplementary Figure 4.** The Nile Red staining procedure does not impact significantly the WMR spectrum of the bacteria interrogated, the peak positions are unchanged and no additional peaks are observed.

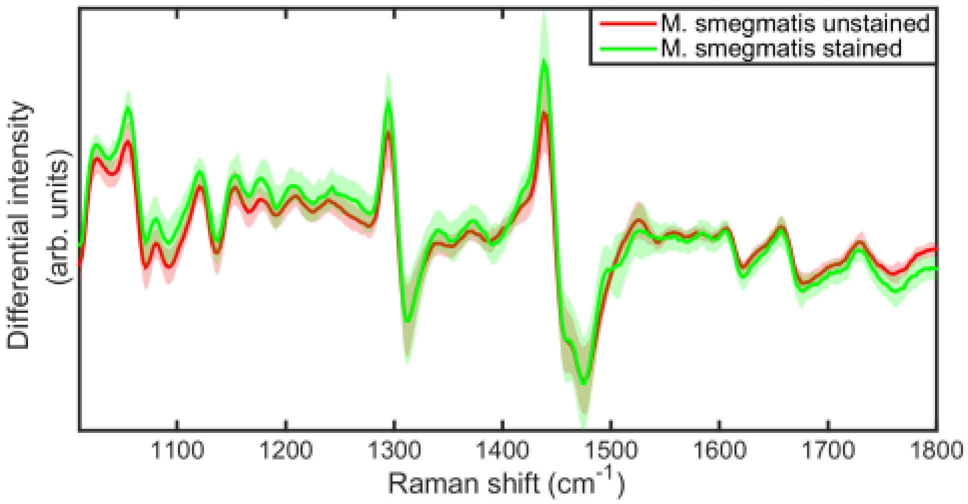

Supplementary Figure 4 *Impact of Nile Red staining on the WMR spectrum of single mycobacterium. WMR spectra of Nile Red stained and unstained M. smegmatis single cells are compared. The coloured lines represent the average spectrum of stained and unstained bacteria in green and red respectively. The shaded area represents the corresponding standard deviation. The x-axis displays the Raman shift in wavenumber (cm^{-1}), the y-axis represents the differential Raman intensity in arbitrary units. The acquisition time per spectrum was 150 seconds.*

References:

1 Hammond, R. J., Baron, V. O., Oravcova, K., Lipworth, S. & Gillespie, S. H. Phenotypic resistance in mycobacteria: is it because I am old or fat that I resist you? *J. Antimicrob. Chemother.* **70**, 2823-2827, doi:10.1093/jac/dkv178 (2015).

2 Ringner, M. What is principal component analysis? *Nat. Biotechnol.* **26**, 303-304, doi:10.1038/nbt0308-303 (2008).

3 Buijtels, P. C. A. M. *et al.* Rapid identification of mycobacteria by Raman spectroscopy. *J. Clin. Microbiol.* **46**, 961-965, doi:10.1128/Jcm.01763-07 (2008).

4 Movasaghi, Z., Rehman, S. & Rehman, I. U. Raman spectroscopy of biological tissues. *Appl. Spectrosc. Rev.* **42**, 493-541, doi:10.1080/05704920701551530 (2007).

5 Maquelin, K. *et al.* Identification of medically relevant microorganisms by vibrational spectroscopy. *J. Microbiol. Methods* **51**, 255-271, doi:10.1016/S0167-7012(02)00127-6 (2002).

6 De Gelder, J., De Gussem, K., Vandenabeele, P. & Moens, L. Reference database of Raman spectra of biological molecules. *J. Raman Spectrosc.* **38**, 1133-1147, doi:10.1002/Jrs.1734 (2007).

7 Wu, H. W. *et al.* In vivo lipidomics using single-cell Raman spectroscopy. *Proc. Natl. Acad. Sci. U.S.A.* **108**, 3809-3814, doi:10.1073/Pnas.1009043108 (2011).

8 De Gelder, J. *et al.* Methods for extracting biochemical information from bacterial Raman spectra: focus on a group of structurally similar biomolecules-fatty acids. *Anal. Chim. Acta* **603**, 167-175, doi:10.1016/j.aca.2007.09.049 (2007).
